# Estimate earth fissure hazard based on machine learning in the Qa’ Jahran Basin, Yemen

**DOI:** 10.1038/s41598-022-26526-y

**Published:** 2022-12-19

**Authors:** Yousef A. Al-Masnay, Nabil M. Al-Areeq, Kashif Ullah, Ali R. Al-Aizari, Mahfuzur Rahman, Changcheng Wang, Jiquan Zhang, Xingpeng Liu

**Affiliations:** 1 Institute of Natural Disaster Research, School of Environment, Northeast Normal University, Changchun, 130024 People’s Republic of China; 2 Key Laboratory for Vegetation Ecology, Ministry of Education, Changchun, 130024 People’s Republic of China; 3 State Environmental Protection Key Laboratory of Wetland Ecology and Vegetation Restoration, Northeast Normal University, Changchun, 130024 People’s Republic of China; 4 Department of Geology and Environment, Thamar University, Thamar, Yemen; 5 Institute of Geophysics and Geomatics, China University of Geosciences, Wuhan, People’s Republic of China; 6 Department of Civil Engineering, International University of Business Agriculture and Technology (IUBAT), Dhaka, 1230 Bangladesh; 7 Department of Surveying and Remote Sensing, School of Geosciences and Info-Physics, Central South University, Changsha, 410083 China; 8 Institute of Surface-Earth System Science, School of Earth System Science, Tianjin University, Tianjin, 300072 China

**Keywords:** Environmental sciences, Natural hazards

## Abstract

Earth fissures are potential hazards that often cause severe damage and affect infrastructure, the environment, and socio-economic development. Owing to the complexity of the causes of earth fissures, the prediction of earth fissures remains a challenging task. In this study, we assess earth fissure hazard susceptibility mapping through four advanced machine learning algorithms, namely random forest (RF), extreme gradient boosting (XGBoost), Naïve Bayes (NB), and K-nearest neighbor (KNN). Using Qa’ Jahran Basin in Yemen as a case study area, 152 fissure locations were recorded via a field survey for the creation of an earth fissure inventory and 11 earth fissure conditioning factors, comprising of topographical, hydrological, geological, and environmental factors, were obtained from various data sources. The outputs of the models were compared and analyzed using statistical indices such as the confusion matrix, overall accuracy, and area under the receiver operating characteristics (AUROC) curve. The obtained results revealed that the RF algorithm, with an overall accuracy of 95.65% and AUROC, 0.99 showed excellent performance for generating hazard maps, followed by XGBoost, with an overall accuracy of 92.39% and AUROC of 0.98, the NB model, with overall accuracy, 88.43% and AUROC, 0.96, and KNN model with general accuracy, 80.43% and AUROC, 0.88), respectively. Such findings can assist land management planners, local authorities, and decision-makers in managing the present and future earth fissures to protect society and the ecosystem and implement suitable protection measures.

## Introduction

Earth fissure is a ground surface rupture phenomenon caused by stress deformation that often occurs in dry and semi-dry basins. Earth fissure develops as fragile subsurface crack. Some of these fissures may be hidden under the surface, while others may become visible on the surface^1–3^. Surface cracks open slowly over time; however, fissures tend to open markedly faster after major rainfall due to soil erosion outside and within the cracks. Earth fissures can be greater than 3 m in width and expand to 10 m or more^1^. Due to land subsidence induced by groundwater extraction, earth fissures are a recurrent problem in Arizona^4^. Arizona’s structural basin contains several long earth fissures (up to 15 km) and short earth fissures (in most cases, a couple of hundred meters)^3,4^. Earth fissures are a long-standing global issue, especially between the 1940s and 1950s^5^. In recent decades, earth fissures have affected several nations, including the US^6–10^, Mexico^11,12^, China^13–16^, India^17^, Iran^18,19^, Saudi Arabia^20^, Pakistan^21^, Ethiopia^22,23^, and Japan^24^. The earth fissures occur in many areas in Yemen, for example, Dhamar city (Jahran Basin and Duran-Anis), Sana'a city (Sana’a Airport and Khawlan), Ma'arib city (Sarwah), Sadaah city (banyhashish), and Abyan city, and it is responsible for many environmental problems. It's worth noting that the earth fissures in Yemen have never been studied previously. Earth fissures result from numerous occurrences, including tectonic activities (e.g., earthquakes, fault movement, and landslides) and human activities (e.g., groundwater withdrawal in dry and semi-dry areas and underground mining)^18,25–30^. Water overpumping can produce substantial underground stress and is considered a key cause of soil compaction^20^. Stress results in large-scale subsidence of the surface and earth fissure^20^. Earth fissure displacement due to groundwater depletion often matches the direction of pre-existing tectonic faults^20,31^. Due to climate change, population growth, urbanization, manufacturing, and drained agricultural operations, expanding groundwater pumping and prolonging the normal recharge period annually are crucial^32–34^. Very few studies on earth fissures hazards mapping have been reported. Budhu ^35^ created a practical mathematical model built on the Mohr–Coulomb failure criterion to explain how earth fissures develop in response to a decrease in groundwater level. Peng et al.^36^ performed a large-scale physical simulation to study the cracking patterns of earth fissures induced by a subsurface fault. Ye et al.^37^ developed a new numerical analysis technique based on interface elements to simulate the formation and propagation of earth fissures in terms of opening earth discontinuities and sliding. By combining the analytic hierarchy process (AHP), the certainty factor model (CFM), and the area under the curve (AUC), Zang et al.^38^ established a probabilistic method for mapping earth fissure hazards. Zhang et al.^39^ evaluated the event of earth fissure using a combination of artificial neural networks and genetic algorithms. Wu et al.^40^ developed a nonlinear modeling and predicting method for earth fissures by combining artificial neural networks and GIS. Choubin et al.^34^ introduced novel ML models for predicting earth fissure hazards and determining critical factors and the impact of human activity. Several scientists have used a planar diagram to depict the relationship between earth fissures and control factors (i.e., earthquakes, faults, and groundwater pumping), ignoring direction and assuming they are scattered across the rock bed region^3,41^. Other scientists recognized that the earliest date (November 13, 1927) of earth fissures was very similar to the earthquake on September 11, 1927, and the Arizona Tree Ring Laboratory photographs recorded^3,32^. Recognizing the controlling factors in earth fissuring is important for enhancing the knowledge of the fissuring process and establishing a reduction strategy for earth fissuring, thereby reducing the danger to the local area. However, it is unknown how these conditions influence the initiation and development of earth fissures^42^. These fissures are typical geohazards that can destroy buildings, farmland, roads and bridges, high-speed rail, subways, gas and oil pipelines, and water supplies, and offer a route for surface contaminants to access and pollute groundwater^12,34,36,43–46^; therefore, global attention has been garnered by earth fissures^37^. Researchers currently use machine learning (ML) techniques in different aspects of geohazard research (e.g., snow avalanches, floods, droughts, gully erosion, landslides, etc.), which is considered a recent breakthrough in the use of ML due to the availability and utility of a wide variety of datasets for ground, environment, atmosphere, and remote sensing (e.g., airborne, space-borne, terrestrial, etc.).

ML algorithms have typically been recorded to surpass classical models in terms of time, precision, intensity, and cost of computational analysis^34,47^. ML also performed well in hazard susceptibility assessment, considering a highly variable frequency and a good sensitivity evaluation^34^. Although the application of artificial intelligence has been used to build perfect models that assess earth fissures, predict landslide susceptibility, map susceptibility to ground subsidence maps, gully erosion vulnerability, and many other geohazards^34,48–53^, the use of ML to map the earth’s fissure hazard has seldom been emphasized. However, there is a lack of agreement on the best approach for mapping vulnerable hazardous areas, particularly in data-scarce nations such as Yemen. Since 2006, earth fissures have occurred throughout the study area, destroying life, properties, and the natural ecosystem. But no attempt has been made to map the risks associated with earth fissures. The study aimed to identify the susceptible locations for earth fissure hazards and the affecting and contributing factors (human and tectonic activity) that affect earth fissure hazards. To address these issues, The objectives of this research are to assess the efficiency of using machine learning models in combination with geospatial techniques for predicting and evaluating earth fissure-prone areas, as well as to identify the major factors that influence the occurrence of earth fissures in the Qa' Jahran Basin. To the best of our knowledge, the XGBoost, NB, and KNN algorithms have not been utilized to investigate earth fissure hazards. As a result, using these algorithms could be seen as a viable means of predicting earth fissure threats.

Additionally, the results may demonstrate the efficacy of the ML approach in detecting the geographical distribution of earth fissures. In this study, the first step was to check the multicollinearity in the conditioning factors and then identify the most important factors accountable for earth fissures occurrence. The next step was to map earth fissure susceptibility mapping using four machine learning models. In the last step, we validate our model, compare four models, and select the best model for the earth fissure in the study area. To the best of our knowledge, this is the first study to attempt earth fissure assessment using ML in the Qa’ Jahran Basin. Therefore, the results of this study will be of great significance for the management of earth fissures in the study region.

## Materials and methods

### Study area

**Figure 1:**
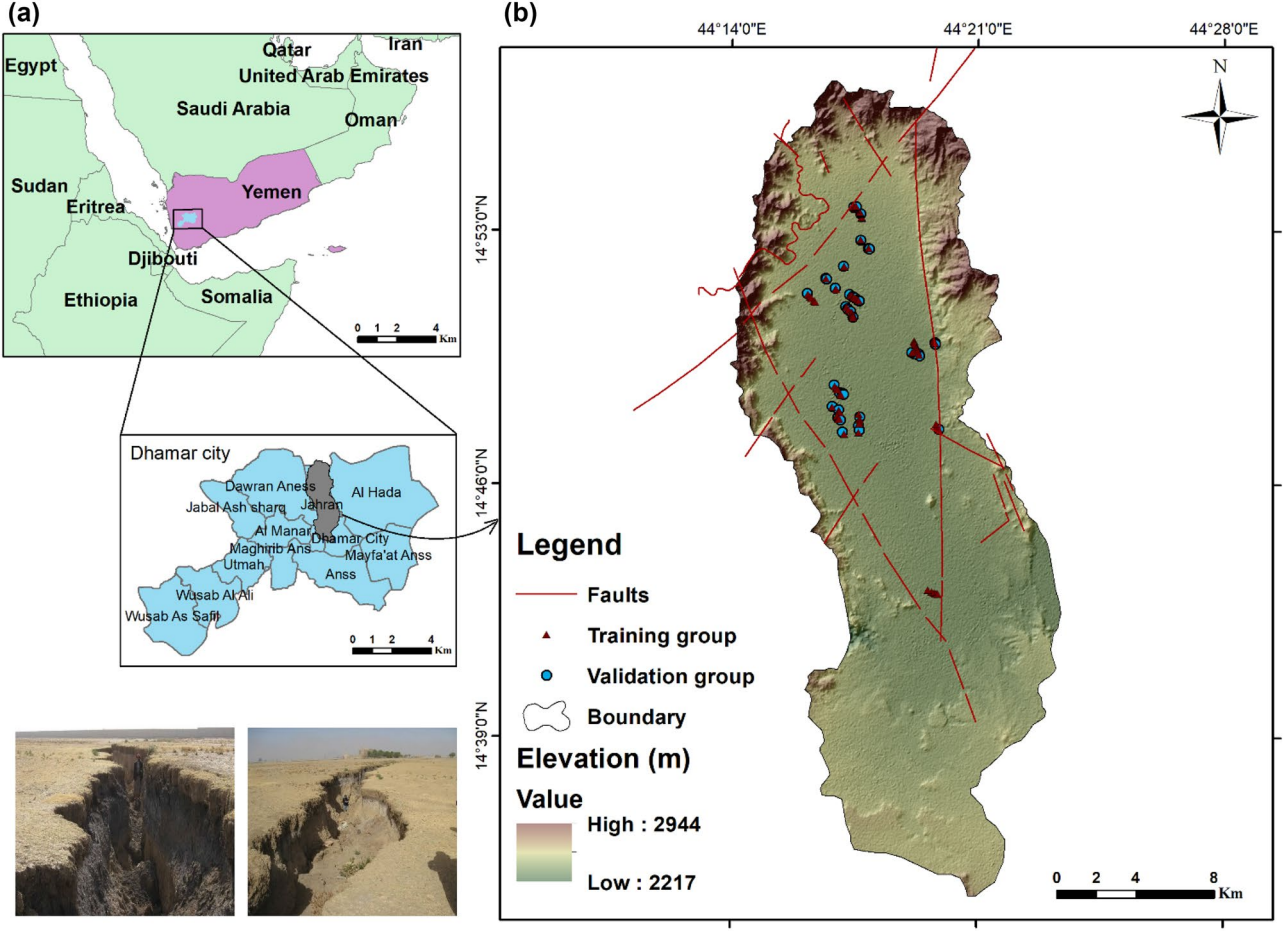
Study area location (Developed by the first author using ArcGIS (v. 10.3), https://support.esri.com, Digital elevation model (DEM) was extracted from ALOS PALSAR DEM https://search.asf.alaska.edu.

The study area is located in the west of Yemen, in Ma’ber District. The basin is one of the most important basins, covering an area of about 413.22 km^2^, and is located between longitudes of 44°12 20″ and 44°22 30″ E and latitudes of 14°38 11″ and 14°57 30″ N. The area lies about 25 km north of Thamar City (Fig. 1). The basin level is between 2304 and 2569 m above mean sea level (m.s.l). As Yemen is generally considered an arid and semi-arid region, such conditions affect the region’s climate, mainly through rain and heat^54^. Rainfall is mainly rare because the basin is surrounded by a chain of high mountains that hinder the access of marine air masses, which generally do not exceed 400 mm. Further, the average temperature varied from 13 to 28 ℃ during 1999–2016 (Dhamar Agricultural Research Center in 2016). The region is also considered to be within the tectonically active zone^54^, where groundwater is the primary water supply for drinking and agricultural purposes. As a result, land subsidence and earth fissures are exacerbated by tectonic movement and groundwater drawdown, causing many subsequent problems.

### Geological aspects of the study area

**Figure 2:**
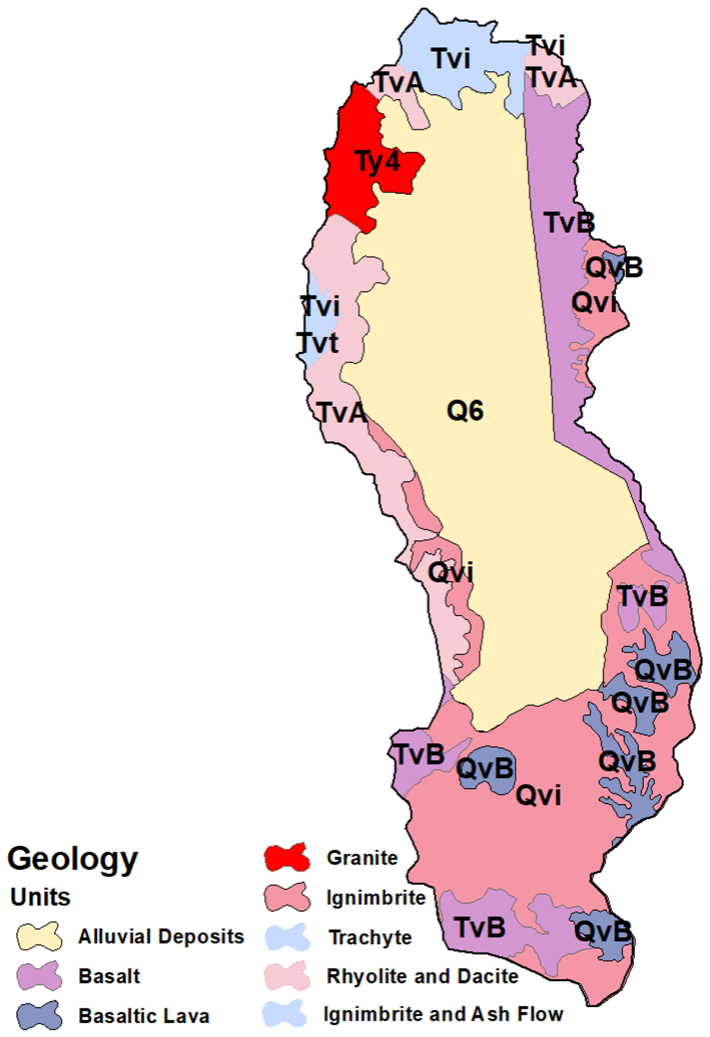
Geological map of the study area (Developed by the first author using ArcGIS (v. 10.3), https://support.esri.com, geological map digitized from the Dhamar geological map obtained from the Yemeni Geological Survey, https://ygsmrb.org.ye/ (free available).

The geology of the area is split into two vast regions, occupied mainly by alluvial deposits and the continental magma area from the Paleogene and Neogene ages, which erupted in the Gulf of Aden and the Red Sea owing to tectonic activity. Large amounts of basalt floods were placed on the west edge in distinct eruptive areas of the Arabian plate from the Mediterranean to the Gulf of Aden in the Oligocene–Miocene^54,55^. In the peripheral region of the catchment area, continental magmatism of varying volume and composition was split into Yemen volcanic series (YVS) and the oldest Yemen trap series (YTS). The YTS formed the lower section of the volcanic Qa’ Jahran Basin and evolved from the Oligocene to Miocene (31–26 Ma)^56^, which was connected to the Afar plume that influenced the Arabia-Africa zone during the Oligocene, and to the opening of the Red Sea and the Gulf of Aden. The YTS occurs in intrusions and lava flows in the study area and comprises ignimbrite, dacite, rhyolite, basalt, trachyte, granite, and ash flow^55^. The YTS also forms a range of semi-steep mountains (within 60°) toward the study area's northern, eastern, and western borders. The northern and western sections of the study area had the highest heights of these mountains. They also outcrop at the western boundary of the pilot area and extend to the south^55^. According to the geological map, there was a significant amorphous granite intrusion in the northwestern part of the study region (Fig. 2). The age of this intrusion is equivalent to that of the Tertiary Granite Invasive of Jabal-Bura (Hodeidah), Jabal-Hufashash (AL-Mahwait), and Jabal Saber (Taiz). This intrusion also has a strong relationship with the opening of the Red Sea Rift structure. Based on geochemical and geochronological evidence, YVS was initially cited by Mattash^57^, Beydoun, et al.^58^. The stage after drift (Miocene to Recent) was created, developed, and divided by an unconformity. The allocated TVS age varies between 11.3 and 0.04 MA^55,57^. The YVS was primarily found in the southeast and south of the study region, with small parts in the western part of the study area. Basaltic lava was placed from a significant normal fault on a considerable mass of Ignimbrite-trending NS. In the study area, the quaternary deposits are shown as plains of quaternary loss sediments in the lengthened depressions found in the study area. These deposits are silt, clay, sand, gravel, alluvial and terraces, and basin alluvium. Alluvial quaternary deposits shape dense cumulations in the center of the region, are eroded toward the margins of the basin, and are powerfully deformed and uplifted east of the basin by a normal fault. Half-graben displacement formed the basin structurally, which resulted in an elongated basin bounded to the east by a natural fault^55^.

### Methods

**Figure 3:**
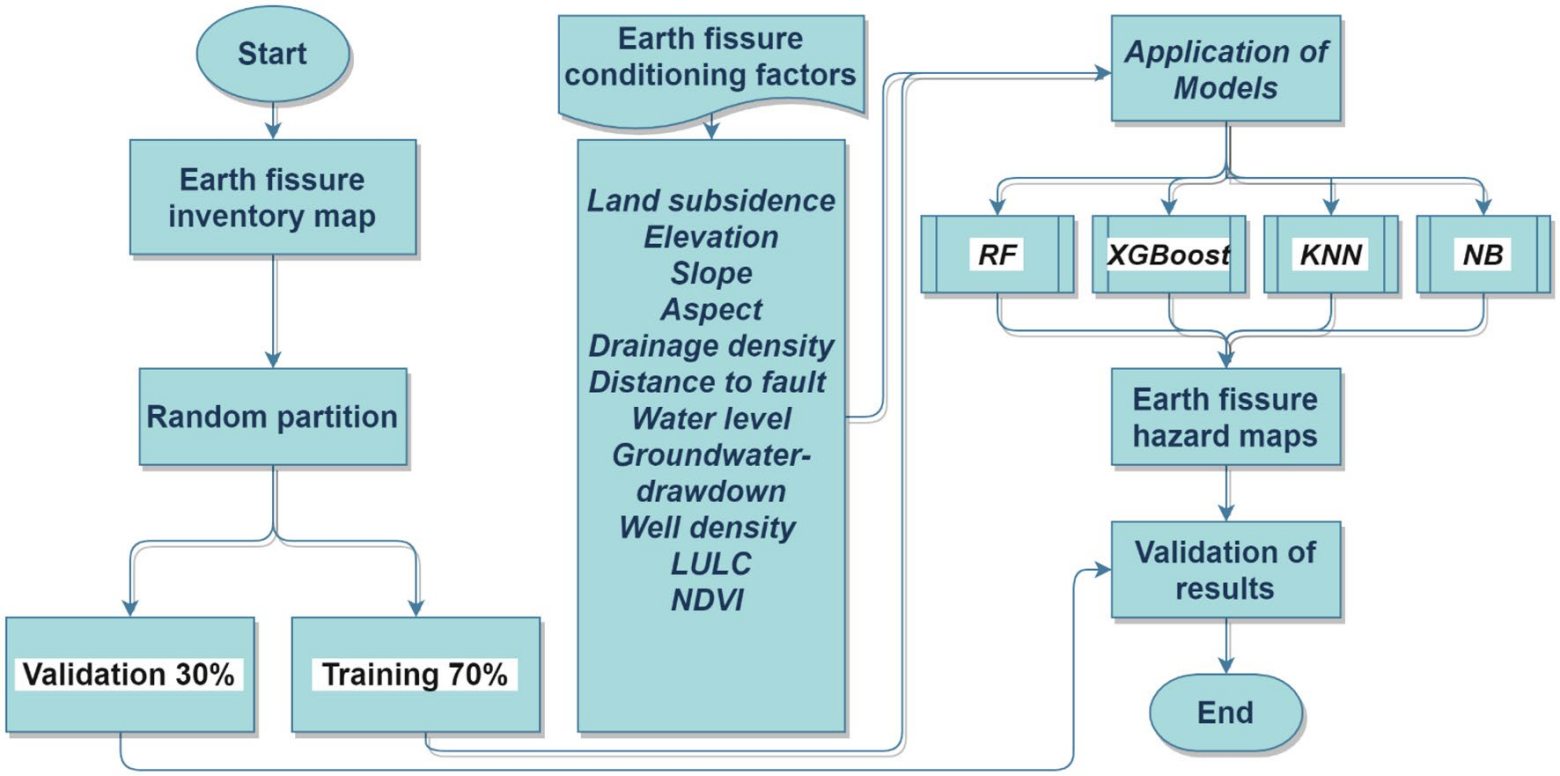
Methodology flowchart (Developed by the first author using draw.io (v. 14.9.6), https://www.diagrams.net/).

The flowchart in Fig. 3 shows the overall procedure followed in this study to set and implement the planned ML models. The workflow can be summarized in three main steps: (1) preparation of input variable thematic layers and earth fissure inventory, (2) modeling of earth fissures using RF, XGBoost, NB, and KNN algorithms, and (3) validation and comparison of the models.

#### Fissure inventory map creation

The construction of an event inventory map is the first and most significant stage in modeling^59^. A total of 152 fissure points (values 1) and 152 non-fissure points (values 0) were collected from the field. Thereafter, 70% of the datasets were used for training and 30% for model validation^59–61^. The extent of data collection and how to divide the data into appropriate data subsets while avoiding over-fitting problems are difficult to determine; however, the survey and data collection were justified to be reasonable and comprehensive for the study area (Fig. 1).

#### Preparation of earth fissure conditioning factors

**Figure 4:**
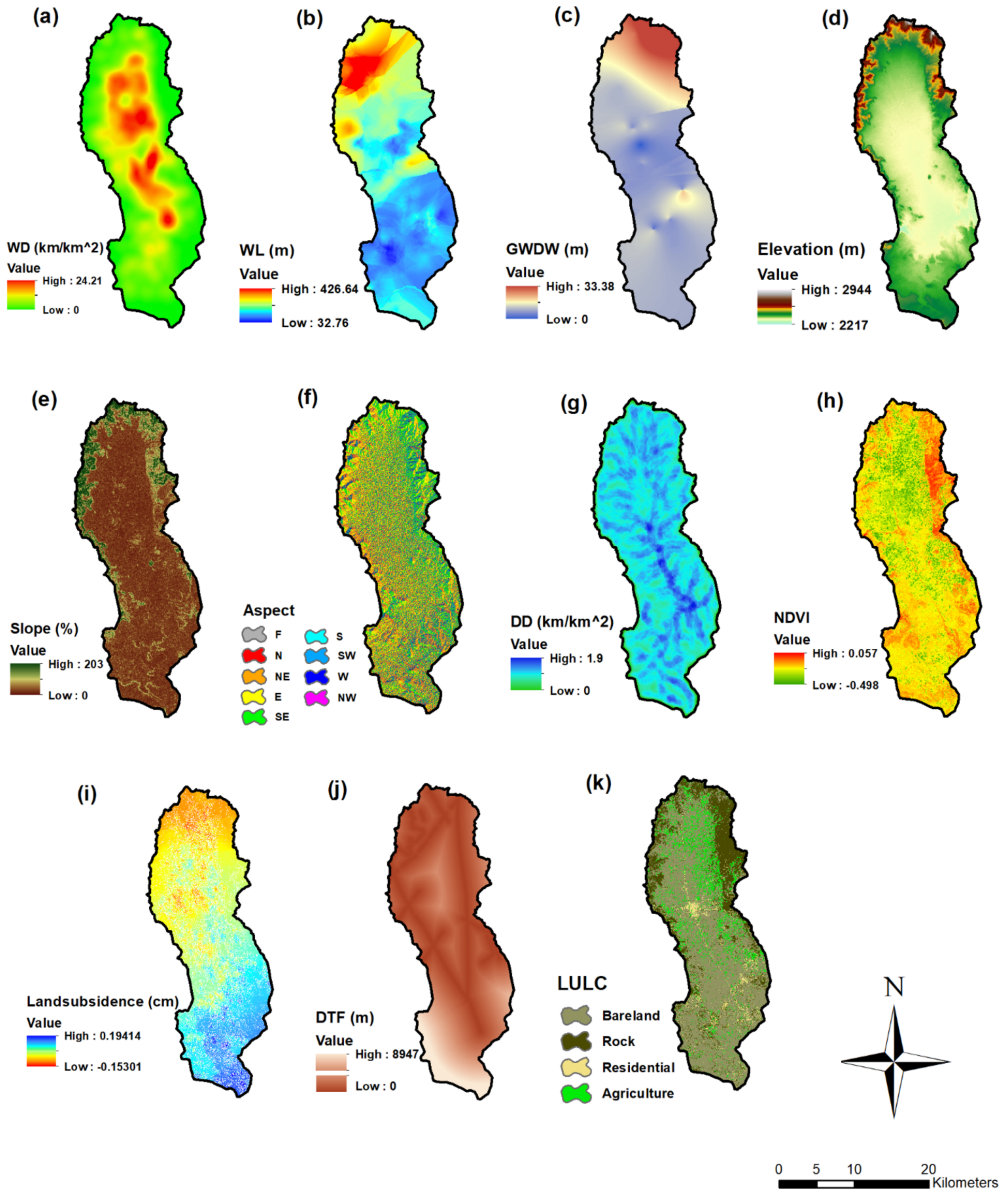
Earth fissure conditioning factors (Developed by the first author using ArcGIS (v. 10.3), https://support.esri.com).

**Figure 5:**
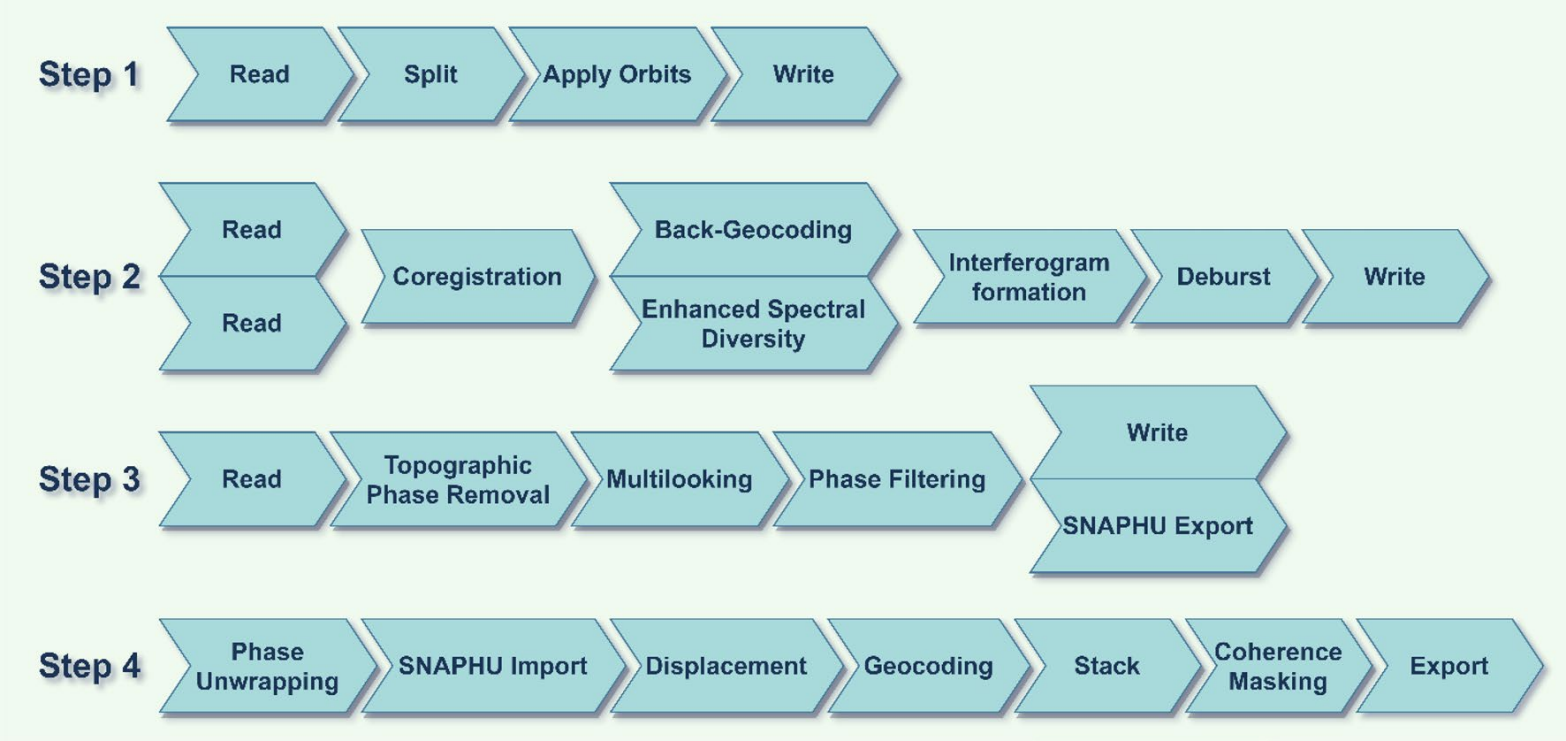
Schematic depicting the various chains of data processing of Sentinel-1 (InSAR) (Developed by the first author using draw.io (v. 14.9.6), https://www.diagrams.net/).

A number of human and environmental factors influence earth fissures. The major factors that influence the event of earth fissures are the extraction and decline of groundwater^1,34,39^. Other significant human activities include land-use changes, and vegetation cover deterioration, all of which might influence the occurrence of earth fissures. Furthermore, environmental factors and topographical factors such as faults, drainage density, elevation and lithology can influence the occurrence of earth fissures. Based on the environmental characteristics and availability in the study region, eleven essential factors were considered in this study (Fig. 4), namely, well density (WD), water level (WL), groundwater drawdown (GWDW), elevation, aspect, slope percentage, drainage density (DD), normalized difference vegetation index (NDVI), land subsidence (LS), distance to faults (DTF), and land use. Elevation, slope, and aspect were extracted from ALOS PALSAR DEM (12.5 m) using ArcGIS (v. 10.3) software (https://search.asf.alaska.edu/). Factors related to drainage density and well density were prepared using the line density tool in ArcGIS (v. 10.3). The local geological map and faults were digitized from the Dhamar geological map obtained from the Yemeni Geological Survey with eight geological units^55^. Distance to fault was calculated from fault lines using the Euclidean distance Tool in ArcGIS (v. 10.3). NDVI and land use maps prepared from Landsat 8 images and Google Earth for June 29, 2020 (https:// earthexplorer.usgs.gov/). Groundwater data were obtained from 20 National Water Resources Authority (NWRA) observation wells. Groundwater drawdown and water level maps were prepared based on 12 years (2008–2020) at observation wells using ArcGIS v. 10.3 (kriging interpolation using Spatial Analysis Tool). Land Subsidence was identified from January 2020 to April 2020 using InSAR (Interferometric Synthetic Aperture Radar) from Sentinel-1 data using the ESA SNAP Toolbox. The data processing steps are shown in Fig. 5^62,63^.

#### Multicollinearity assessment of conditioning factors

The multicollinearity test may improve model results in natural hazard studies by selecting ideal factors for hazard mapping^64^. Multicollinearity refers to the absence of independence of the independent variables and their significant correlations, which can arise in a dataset and mislead an analysis of their incidence^65^. To examine the multicollinearity of independent variables in earth fissure modeling, the tolerance (TOL) and variance inflation factors (VIF) were used in this study^66^. A multicollinearity problem is indicated by a VIF score of 10 or above and a TOL of less than 0.10^67^:

1 $$Tolerance=1- {R}_{j}^{2}\frac{TP+TN}{TP+TN+FP+FN}$$

2 $$VIF=\left[\frac{1}{Tolerance}\right]$$

where ${R}_{j}^{2}$ is the coefficient of determination.

#### Applied ML models for earth fissure hazard mapping

To predict earth fissures, the machine learning supervised classification techniques used in CARET packages provided by R. CARET packages provide functions for preprocessing, model training, model prediction, and model evaluation. Once finished preparation of the dependent and independent factors, the dataset (training and testing data) was imported to R. The preprocessing for the dataset encodes the categorical variable (and scaling) as a set of boolean inputs, each representing one category with 0 or 1. After that procedure, the perfect split between predicting which variable would be the best for splitting the decision tree and visualization data to see the relationship between the variables and earth fissure frequency.

**Figure 6:**
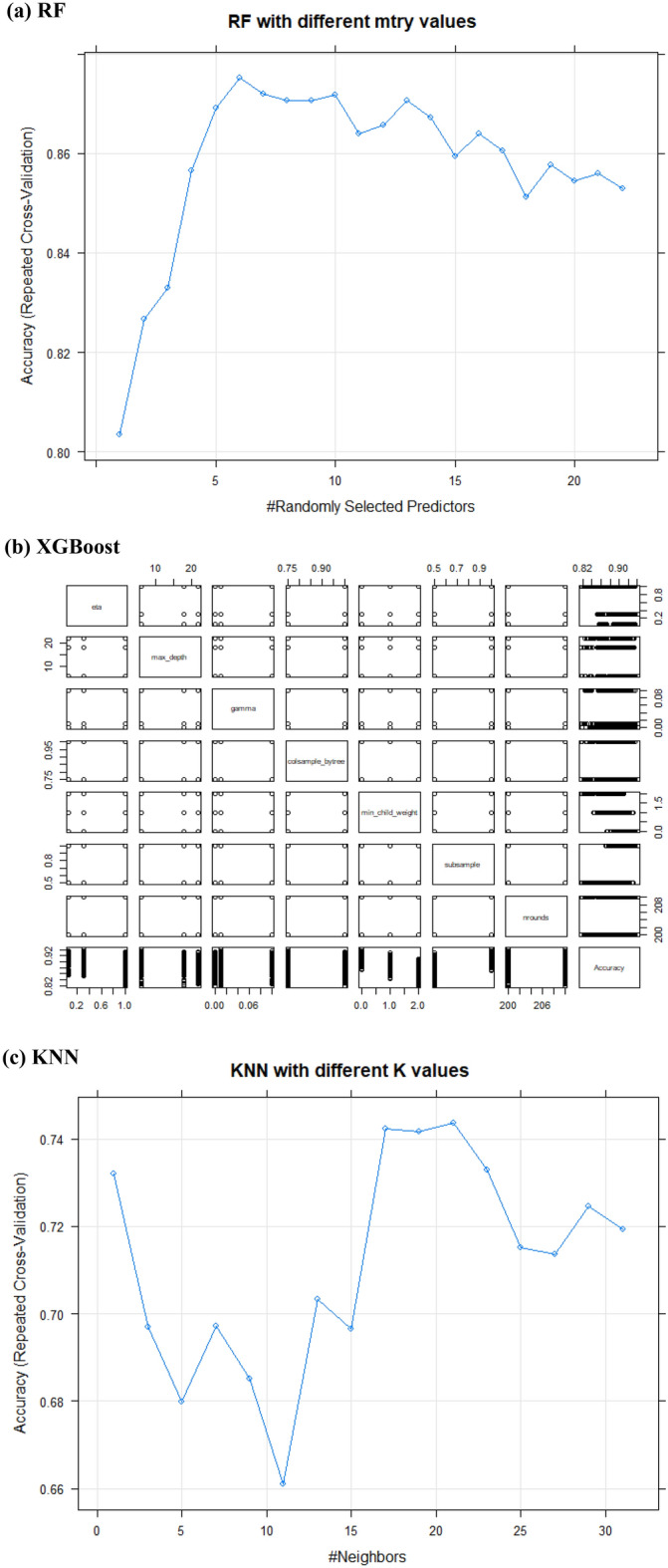
The relationship between each hyperparameter with accuracy (Developed by the first author using R studio software (v. 3.6.1).

RF, XGBoost, NB, and KNN models were proposed using all datasets with the best conditioning factors. Models were developed to operate with default settings. Hence, hyperparameters were optimized with multiple values and re-run with recommended tuning parameters that gave us the highest accuracy (Table 1). The RF algorithm used 500 trees, and the model’s best final value was mtry = 7, with a better grid search than random search (Fig. 6a). In the XGBoost algorithm, subsample, min child weight, and eta were found to have a clear enhancement in accuracy. In particular, when the sub-sample produced better accuracy when reaching a value of 1, for minimum child weight produced better accuracy with the value 0 more than the values of 1 and 2, also eta produced the best accuracy with the value of 0.3 more than the values of 0.05 and 1. nrounds, colsample-bytree, gamma, and max-depth gave better accuracy with the values of (200, 0.75, 0.01,6) respectively. Still, there was not that effective accuracy if change the values (Fig. 6b). The NB algorithm is used to expand. Grid (FL = c (0), usekernel = T, and adjust = c (0.5) to achieve the highest accuracy (Fig. 6c). There was not much difference in accuracy between default parameters and tunned hyperparameters. Therefore, we used the parameters default setting because it was less time-consuming. In the KNN model, the default parameters and tunned hyperparameters agree on the value of k = 21; when the value of k increases, the start accuracy decreases again. Of note, optimizing hyperparameters is a critical step in maximizing accuracy efficiency. After that, run the confusion matrix to evaluate the model, plot ROC curves to calculate the values of AUC, and then produce a prediction map using raster data.

**Table 1:** Recommended settings for the hyperparameters.

Model	Recommended settings	
RF	mtry	7
	ntree	500
	Repeats	3
	Search	Grid
XGBoost	eta	0.3
	Max depth	6
	Gamma	0.01
	colsample bytree	0.75
	Min child weight	0
	Subsample	1
	nrounds	200
NB	FL	0
	Usekernel	T
	Adjust	0.5
KNN	K	21

##### Random forest

RF is an ensemble learning algorithm designed to improve the regression and classification of trees by integrating a wide range of decision-making trees^68^. RF is an effective method for managing data vagueness and complexity and has been successfully used to evaluate many complex datasets^50,69,70^. Owing to its robustness, flexibility, and manageability of complex data structures, RF has also been proven to be one of the best-used hazard modeling techniques^71–74^. The RF algorithm has two solid techniques: random subspace collection and bagging^75^. RF produces binary tree (ntree) classifications using bootstrap samples to replace the raw values. These classification trees participate to unit voting, and the proper classification is a consensus vote for all forest trees. Three key parameters employed in the implementation of the RF technique are the number of trees (ntree), the number of acceptable characteristics for splitting (mtry), and the minimum number of observations in terminal node (node size) (). In this literature, you can find further mathematical details^68,76^.

##### Extreme gradient boosting

Compared to other algorithms, the XGBoost algorithm has received extensive attention owing to its superior efficiency, excellent learning impact, and efficient training speed^77,78^. The XGBoost algorithm is a gradient-boosting decision tree (GBDT) enhancement technique that is useful for solving regression and classification tasks. XGBoost is a boosting tree algorithm that combines many weak classifiers into a robust classifier. This algorithm works by constantly adding trees and dividing the features in order to grow a tree. A new function that matches the last residual predicted can be learned^78^. Three key parameters, sub-sample (sub-sample ratio of training instance), namely colsample bytree (sub-sample ratio of columns when building each tree), and nrounds (max number of iterations boosting), are used in XGBoost^79,80^.

##### Naïve Bayes

NB is a simple and widely used algorithm applied in various fields (computer science, earth sciences, text classification, and medicine)^81^. This approach is practical when sample X can be characterized as conjugating conditionally independent attributes^81,82^. Based on Bayesian probability theory, Bayesian learning enables us to compute the posterior probability given the prior chances^83,84^. The primary advantage of the NB model is that it is relatively simple to implement and does not necessitate the use of extensive hyperparameter tuning^84^. The mathematical foundation of NB is strong, and its categorization efficiency is consistent. NB works well with tiny amounts of data, can handle several categorization jobs, and can be trained incrementally. The disadvantage of the NB model is that it is susceptible to how the input data is represented; it is necessary to compute the prior probability^85^.

##### K-Nearest Neighbor

KNN algorithms are supervised ML algorithms that do not require learning; they are also referred to as lazy algorithms^86^. KNN can be used to handle regression and classification issues^81,85^. KNN computes the k nearest samples utilizing the distance between samples and uses their value to predict the value of the desired sample^81,87^. These k samples are most similar to the sample examined. Once the method has selected the k nearest samples, it may simply output a weighted sum of their values as the model's prediction for the target sample. KNN’s drawbacks include the necessity for extensive calculation and the requirement for large memory^81^.

#### Models testing

Validation is an integral part of the modeling process^50^. Validation is performed in every modeling technique to consider whether the model has achieved reasonably reliable results for the target^88^. Model evaluation helps determine the suitability of the model and the elements that require enhancement^50^. Thus, 30% of the datasets were used to validate the models. The confusion matrix, overall accuracy and area under the receiver operating characteristics (AUROC) curve were considered to validate the earth fissure models in this study.

##### The receiver operating characteristic (ROC) curve and Kappa index

Analysis can be used to evaluate the performance of earth fissure hazard models. The ROC curve is a graph with varying cut-off thresholds depending on Specificity and Sensitivity. AUROC, a statistical overview of the overall performance of the earth fissure models, is utilized for quantitative comparison^89^. The AUROC expresses the likelihood that the classifier would properly rate a randomly chosen earth fissure pixel as more indicative of an earth fissure than a selected randomly non-earth fissure. When AUROC is equal to 0, it suggests a non-informative model, however; however, when AUROC is equal to 1, it represents a great model that correctly identifies all earth fissure and non-earth fissure pixels^89,90^. The AUROC standard error was used to examine the importance of one classified system having a larger AUROC than another^91^. The model will perform better if the standard error is small. The Kappa index (κ) can be used to determine the trustworthiness of earth fissure models^92,93^. The Kappa index is used to quantify the capacity of earth fissure models to classify earth fissure pixels^94^. It is calculated as the ratio of measured agreement that randomly exceeds the probability of this occurring. According to Landis and Koch^95^, the strength of agreement given the Kappa magnitude is ≤ 0 poor, 0–0.2 slight, 0.2–0.4 fair,0.4–0.6 moderate, 0.6–0.8 substantial, and 0.8–1.0 almost perfect^89^.

##### Quality parameters and accuracy measure

Five statistical evaluation measures were employed to assess the performance of the trained earth fissure models: accuracy, specificity, sensitivity, negative predictive value, and positive predictive value. Accuracy is defined as the proportion of fissure and non-fissure pixels accurately detected by the producing model. Specificity is the ratio of non-fissure pixels accurately classified as non-fissure. The ratio of fissure pixels accurately identified as fissure occurrences is called sensitivity. The likelihood of pixels correctly identified as non-earth fissure is the negative predictive value. In contrast, the likelihood of pixels correctly identified as fissure is the positive predictive value^89^.

3 $$\mathrm{Accuracy}=\frac{TP+TN}{TP+TN+FP+FN}$$

4 $$\mathrm{Specificity}=\frac{TN}{FP+TN}$$

5 $$\mathrm{Sensitiviy}=\frac{TP}{TP+FN}$$

6 $$\mathrm{Negative predictive value}=\frac{TN}{FN+TN}$$

7 $$\mathrm{Positive predictive value}=\frac{TP}{FP+TP}$$

where TP (true positive) and TN (true negative) are the numbers of pixels that are correctly identified, whereas FP (false positive) and FN (false negative) are the numbers of pixels erroneously identified.

## Results

### The results of factors multicollinearity analysis

The 11 earth fissure conditioning factors were tested for multicollinearity Table 2. TOL and VIF of earth fissures conditioning factors ranged from (0.237–0.939) to (1.064–4.206), respectively. VIF < 10 and tolerance > 0.1 are satisfied threshold values. As a result, the conditioning factors chosen in this investigation had no multicollinearity. Therefore, all conditioning factors were considered to model the earth fissures and were subsequently analyzed.

**Table 2:** Multicollinearity of earth fissure conditioning factors.

Variables	Collinearity statistics		Variables	Collinearity statistics	
	Tolerance	VIF		Tolerance	VIF
Distance to fault	0.7463	1.3399	Slope	0.4291	2.3305
Drainage density	0.7280	1.3737	Water level	0.4142	2.4143
Elevation	0.2377	4.2063	Well Density	0.4463	2.2404
Groundwater drawdown	0.3948	2.5331	Aspect	0.9399	1.0640
Land subsidence	0.5607	1.7834			
Land use	0.8216	1.2171			
NDVI	0.6203	1.6121			

### The results of earth fissure hazard mapping

**Figure 7:**
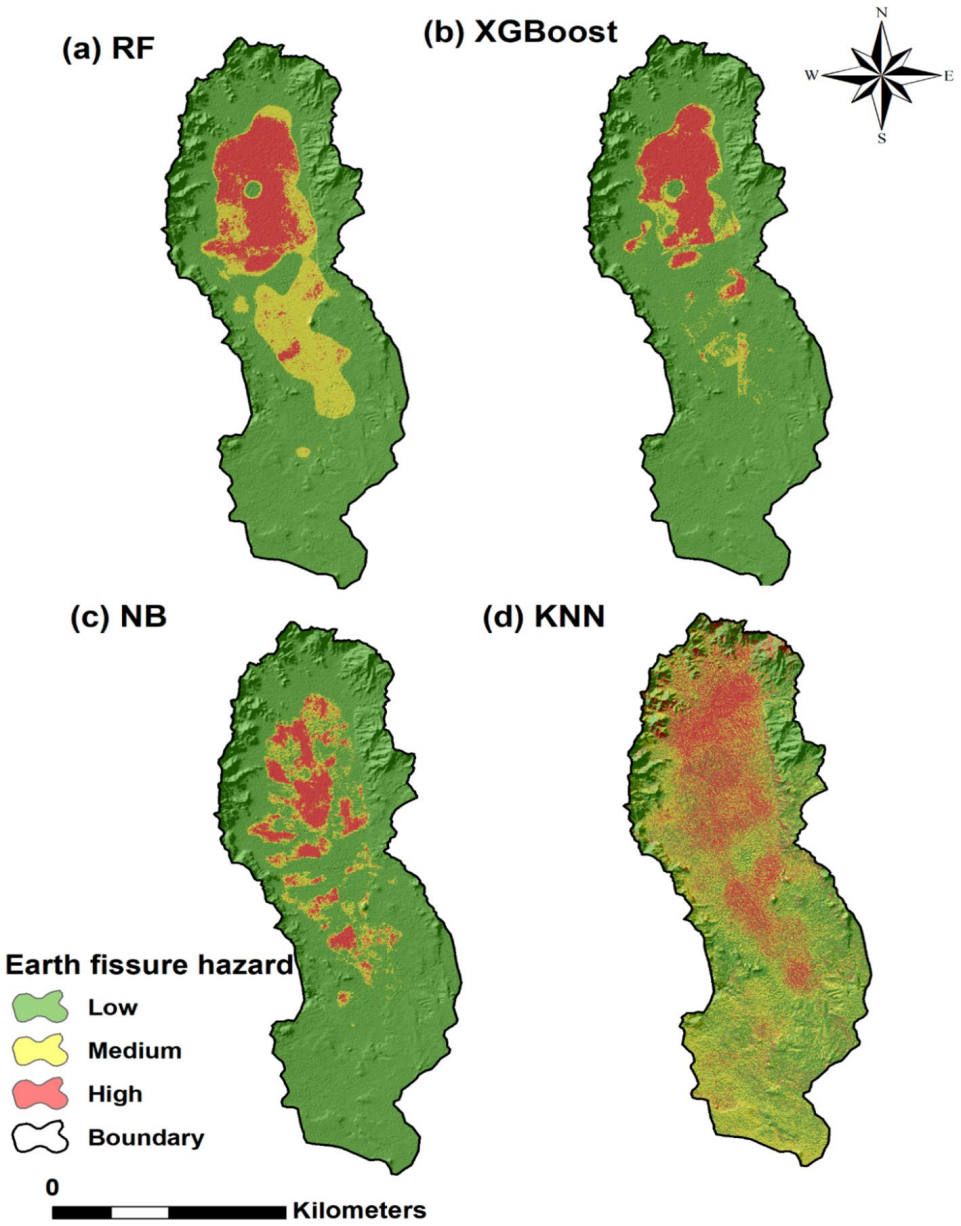
Maps of earth fissure hazard by the (**a**) RF, (**b**) XGBoost, (**c**) NB, and (**d**) KNN models (Developed by the first author using ArcGIS (v. 10.3), https://support.esri.com).

**Figure 8:**
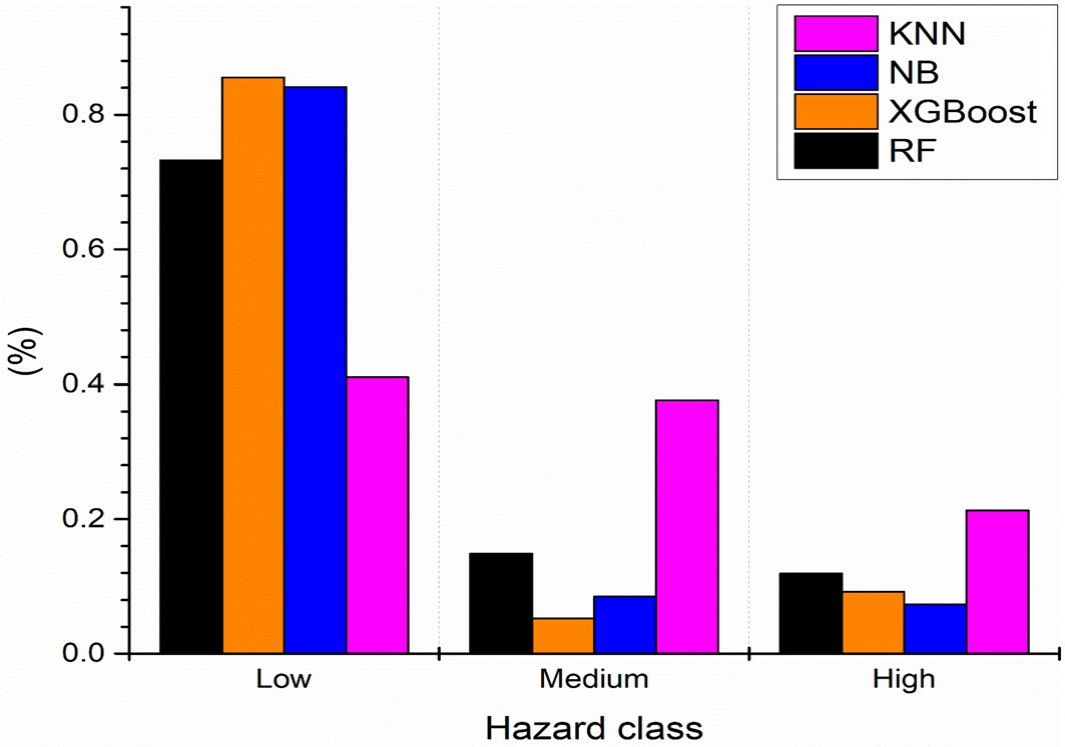
Percentages of the three-earth fissure hazard class (Developed by the first author using graphpad (v.9.2.0) https://www.graphpad.com).

In this study, the ML models have been used to assess and map earth fissure hazard; all the models were built in R studio software packages (version 3.6.1). After applying the training dataset for the RF, XGBoost NB, and KNN models, earth fissure hazard indices were calculated for all parts of the research area^34^. After applying the training dataset for the RF and XGBoost, NB, and KNN models, earth fissure hazard indices were calculated for all parts of the research area^34^. Earth fissure hazard indices were reclassified into three hazard levels (low, medium, and high) using a similar field classification procedure^34^. According to the percentage of earth fissure pixels and the percentage of earth fissure hazard map, the three hazard classes were identified as low hazard, with values 299.15 km^2^ (73.21%) for RF, 349.47 km^2^ (85.52%) for XGBoost, 343.76 km^2^ (84.13%) for NB, and 167.67 km^2^ (41.03%) for KNN. The medium hazard class for RF, XGBoost, NB, and KNN models, respectively, cover 60.82 km2 (14.88%), 21.48 km^2^ (5.25%), 34.82 km^2^ (8.52%) and 153.89 km^2^ (37.66%) of the total area. Finally, the high hazard class for RF, XGBoost, NB, and KNN models, respectively, cover 48.62 km^2^ (11.90%), 37.64 km^2^ (9.21%), 30.01 km^2^ (7.34%) and 87.03 km^2^ (21.30%) of the total study area. The models predicted that the high hazard would be concentrated in the northern part of the study area (Figs. 7 and 8).

### Validating and comparing the models

**Figure 9:**
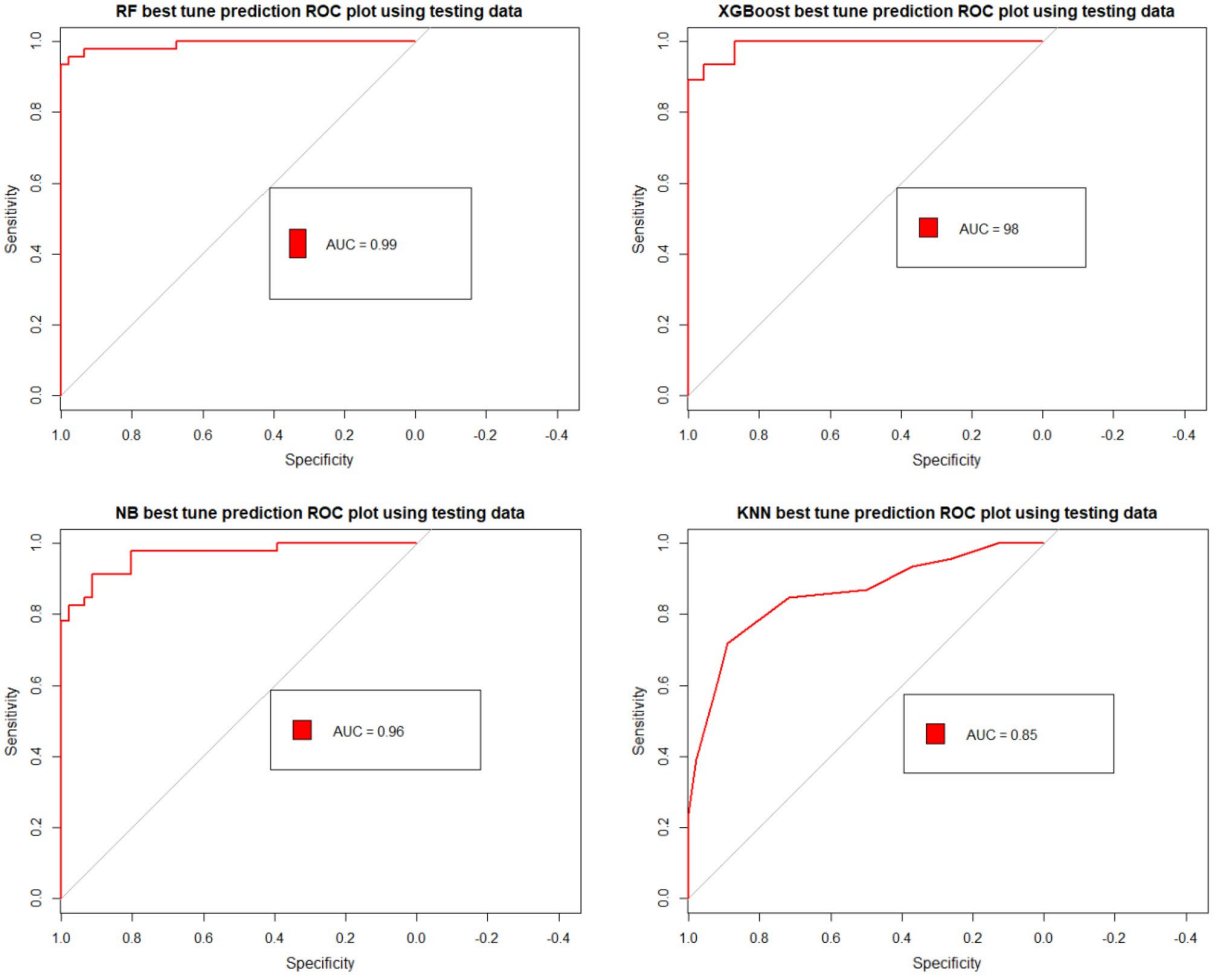
ROC curve showing AUC values of RF, XGBoost, NB and KNN (Developed by the first author using R studio software (v. 3.6.1).

Models were validated with testing data of 30% of the total points using AUROC^59^. This study used the AUROC method due to its correspondence, satisfaction, and ability to produce quantitative model estimates. All the models achieved very good to excellent results, with AUROC values found to range from 88 to 99% (Fig. 9). Considering the AUROC process, there was no significant difference in the output between the RF, XGBoost, and NB models. As presented in (Fig. 9), the RF produced an AUROC of 99% and overall accuracy of 95.6%. However, XGBoost had an AUROC of 98% with an overall accuracy of 92.3%. NB produced an AUROC of 96%, with an overall accuracy of 88%. In comparison, the KNN produced an AUROC of 88%, with an overall accuracy of 80.4%. Hence, the RF, XGBoost, and NB algorithms were proven to achieve better hazard modeling. The achieved consistency between the applied model ensures that the model is sufficiently accurate to predict possible future earth fissures over the region.

The RF, XGBosst, KNN and NB were evaluated by various statistical measures (Tables 3 and 4). RF model with AUC values of 99% achieved the highest accuracy, followed by XGBoost with AUC values of 98%, NB with AUC 96%, and KNN model with AUC values of 88%, respectively. The models demonstrated excellent results in predicting earth fissures hazard in the study area with AUC > 88%. Furthermore, the kappa index was used to assess the reliability of earth fissure models; the kappa value of the KNN model was found to be 0.608, indicating a “moderate” agreement. Also, the kappa value of the NB model was set at 0.760, indicating a “substantial” agreement. Furthermore, the RF (0.913) and XGBoost (0.847) models have achieved a perfect agreement in terms of Kappa value. The Kappa index value indicates model compatibility and reliability; additionally, there is a high degree of congruence between models and reality. In the case of the classification of the earth fissure zone, the highest predictive positive value was observed in the RF model, indicating the model’s likelihood of classifying the earth fissure zone better in 97.83% of situations. Compared with the RF model, the NB model achieved a value of 92.68%, followed by the XGBoost model (88.24%) and the KNN model (86.84%), respectively. Additionally, the XGBoost model achieved the highest negative predictive value (97.56%), which indicates that the likelihood of correctly classifying the non-fissure zone was 97.56%. However, the RF model achieved 93.75%, followed by the NB model (84.31%) and the KNN model (75.93%), respectively. In the case of classification of earth fissure pixels, the XGBoost model produced the highest sensitivity (97.83%), revealing that 97.83% of earth fissure pixels were correctly rated as earth fissures, while the RF model correctly rated 93.48%, followed by the NB model (82.61%), and KNN model (71.74%), respectively. Additionally, the RF model achieved the highest specificity (97.83%), which indicated that 97.83% of the non-earth fissure region was adequately defined as a non-earth fissure. In contrast, the NB model achieved a specificity of 93.48%, followed by the KNN model (89.13%) and the XGBoost model (86.96%), respectively. Overall, four modes of earth fissure achieve better results in the classification of earth fissure and non-earth fissure pixels. Overall, in this study, four earth fissure models are acceptable, and the RF model displays the most stable and efficient results among all models.

**Table 3:** Model performance.

Parameter	RF	XGBoost	NB	KNN	Mean	SD	95% CI
Positive predictive value (%)	97.83	88.24	92.68	86.84	91.4	4.96	91.39 ± 4.85 (± 5.32%)
Negative predictive value (%)	93.75	97.56	84.31	75.93	87.89	9.72	87.88 ± 9.53 (± 10.84%)
Sensitivity (%)	93.48	97.83	82.61	71.74	86.42	11.69	86.41 ± 11.45 (± 13.26%)
Specificity (%)	97.83	86.96	93.48	89.13	91.85	4.82	91.85 ± 4.72 (± 5.14%)
Accuracy (%)	95.65	92.39	88.04	80.43	89.13	6.58	89.12 ± 6.45 (± 7.24%)

**Table 4:** AUROC and Kappa index for the earth fissure models.

Earth fissure models	AUROC	95% CI	Kappa index
RF	99	0.8924, 0.988	0.913
XGBoost	98	0.8495, 0.9689	0.847
NB	96	0.7961, 0.9388	0.760
KNN	88	0.7085, 0.8797	0.608

### Analysis of conditioning factors importance

**Figure 10:**
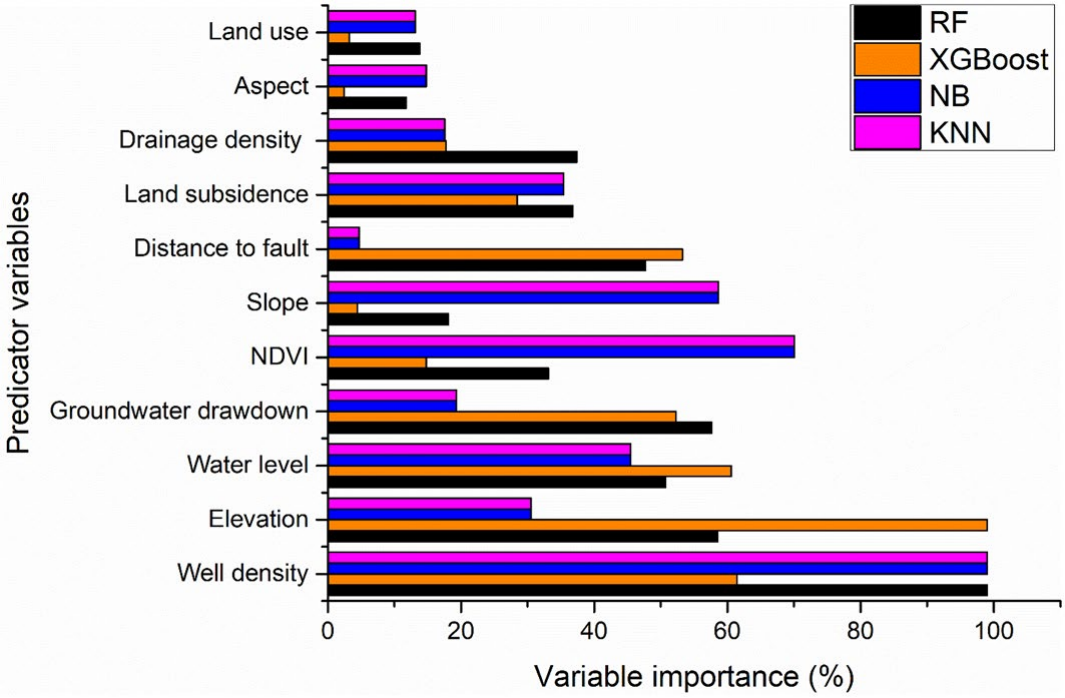
The importance rank of the earth fissure predictors (Developed by the first author using graphpad (v.9.2.0) https://www.graphpad.com).

The sensitivity and significance of every earth fissure conditioning factor are essential outputs used to calculate the earth fissure hazard map^34^. The OOB in the RF was used to rank the significance of the conditioning factor during the model training process (Fig. 10). For the RF model, well density was the most important factor, followed by elevation, groundwater drawdown, water level, distance to faults, drainage density, land subsidence, NDVI, slope, land use, and aspect. The most important factor in XGBoost was elevation, followed by well density, water level, distance to fault, groundwater drawdown, land subsidence, drainage density, NDVI, slope, land use, and aspect. In contrast, the most important factor for NB and KNN was well density, NDVI, slope, water level, land subsidence, elevation, groundwater drawdown, drainage density, aspect, land use, and distance to the fault. In the case of KNN and NB, well density is the most important factor for predicting earth fissures, followed by NDVI, slope, water level, land subsidence, elevation, and groundwater drawdown. Our results are aligned with those of previous studies, demonstrating that excessive water withdrawal, high well density, and high groundwater extraction contribute to earth fissures^3,34,96^. In contrast, aspect and land use were identified as the least important factors. The modelling strategy affects the comparative relevance of the predictor variables to earth fissure modeling^89^. Therefore, for one model, predictive variables of high relative importance may be useless for another model. Thus, in different models, the importance of a predictor variable can differ from each other^89^.

## Discussion

### Evaluation of factors of importance

Evaluating the significance of predictors factors is useful for environmental managers responsible for allocating and planning scarce natural resource management resources^97^. Brown and Nicholls^98^ emphasized the importance of analyzing the relationship among land subsidence, earth fissures, and environmental factors since it enables the planners to focus on human activities’ influence. While numerous analytical and expert opinion-based methods for analyzing natural hazards have been presented, the relative impact of geo-environmental variables continues to be discussed^99^. In general, decision-makers have benefited from fresh insights into the linkages between hydro-geological and geo-environmental factors to ML algorithms, as well as the occurrence of earth fissures, and they are now viewed as a convenient tool capable of effectively contributing to environmental management improvement^100^. The modelling strategy affects the comparative relevance of the conditioning factors to earth fissure modeling. Therefore, for one model, predictive factors of high relative importance may be useless for another model. Thus, in different models, the significance of predictor factors can differ from each other^89^. The comparative relevance of predictors was determined in our study using the RF, XGBoost, NB, and KNN models. The analysis determined that well density is the main factor for predicting earth fissures in RF, NB, and KNN models. These results align with those of previous studies, demonstrating that excessive water withdrawal, high well density, and high groundwater contribute to the issue of earth fissures^3,34,96^. The intense groundwater withdrawal has resulted in a catastrophic fall in potentiometric levels, putting aquifer systems under strain and stress, eventually resulting in earth fissures and land failures^101^. Ground deformation caused by pumping occasionally happens in aquifer systems with poorly cemented sediments^102^. As Burbey^103^ discusses, favourable conditions for land subsidence and earth fissures include the following: (1) long-period groundwater extraction that causes a large drop in the water table, (2) materials that are thick and compressible, and (3) failures of the tectonic plates (e.g., faults) and geological discontinuities that allow for the buildup of stress. Thus, excessive groundwater extraction should be severely restricted in the majority of the study area to prevent the development of earth fissures. Additionally, artificial recharging of groundwater (ARG) initiatives improve an aquifer's water balance, potentially reducing the hazards of earth fissures. In all four models, the aspect and land use were recognized as the least important factors.

### Predictive performance of models

Modeling and simulation can help us learn more about environmental threats and make better decisions. The structure of modeling methods, on the other hand, varies significantly, resulting in a wide range of outputs and predictive performance. Model-based spatial predictions are presently recognized as a critical aim of ecological and geo-environmental research since they will guide managers’ and environmental planners’ decision-making^104^. Notably, the diversity of modeling techniques allows planners to become conscious, comprehend and build effective environmental plans^105^. According to Araujo and Guisan^106^, even while the same model types are used in various sectors, there may be heterogeneity in the forecasts and results. Thus, comparative studies are necessary to analyze models’ performance in similar environments and accurately appraise their abilities^107^. This work explored four machine learning approaches (RF, XGBoost, NB, and KNN) to determine the most accurate way to assess earth fissure locations. The RF model (AUROC = 99%) overcomes the XGBOOST (AUROC = 98%), NB (AUROC = 96%), and KNN (AUROC = 88%) models in terms of AUC values. There was no discernible difference in predictive performance or goodness-of-fit between models. The RF model outperforms the XGBoost, NB, and KNN models in terms of performance. The KNN method predicted that most central areas have a medium or high occurrence of earth fissures. In contrast, the RF, XGBoost, and NB models classified these areas as having a lower occurrence of earth fissures. In general, the four models agreed to predict most hazard areas. Moreover, the high-hazard areas align with where earth fissures occur in the basin. The achieved consistency between the applied model ensures that the model is sufficiently accurate to predict possible future earth fissures over the region. Few research studies have been conducted to investigate the performance of RF, XGBoost, NB, and KNN models in geo-environmental fields (e.g., landslide, air quality, and flash flood)^80,86,108–111^. However, it is difficult to directly compare our findings to this research because XGBoost, NB, and KNN models were not previously utilized to predict earth fissures. Therefore, our models’ performance was compared with the same models in other hazard assessment applications. High-accuracy models have been highlighted in the literature^110,111^, and they found RF to be the most successful model. This outcome is in line with what we found in our research. KNN was also found to be as effective^112^. Naghibi et al.^111^ also emphasized the KNN model's higher accuracy. In a study comparing the performance of ML algorithms for flood susceptibility prediction, Madhuri et al.^113^ found that XGB outperformed KNN and that RF, XGBoost, and NB outperformed KNN as well. Although it had the lowest predicted accuracy of the three ways evaluated, the KNN method was similarly beneficial. Of note, the results obtained in this study were found to coincide with similar work on the exact application nature^34^. They introduced a compared several models for predicting earth fissure hazards. They found the RF model best predicted the earth fissure hazard. Notably, both tree-based (RF and XGBoost) models performed well to well, demonstrating their overall capability for modeling earth fissures. According to França et al.^105^, whereas linear modeling approaches usually fail to satisfy a variety of statistical assumptions, such as variable independence and variable statistical distribution, tree-based models frequently escape these limits.

Tree-based ML models, notably RF and XGBoost, were shown in this work to be capable of uncovering complex nonlinear relationships. These results are consistent with similar findings in^84^, which provided an extensive comparison of various ML models, where it was found that tree-based models are superior to other ML models. As it turned out, the fundamental drawback of single-tree models was overcome by fitting multiple trees in RF, and XGBoost models^114,115^. As a result, based on the models’ performance and ease of interpretation, this study shows that the chosen models are genuinely possible. Advanced environmental hazard analyses are needed as the increasing human population leads to high demand for shelters and infrastructure. Further accurate studies are required in order to identify hazard-free zones.

## Conclusion

In this study, we used four ML algorithms (e.g., RF, XGBoost, NB, and KNN) to model and forecast earth fissure hazard levels and classify the key processes leading to the hazard in the Qa’ Jahran Basin, Yemen. The results show that approximately 7.34–21.30% of the overall area was found to be highly vulnerable to earth fissure hazard, 5.25–37.66% to a medium hazard level, and 41.03–85.52% to low hazard level. The region’s most sensitive to earth fissure hazards were found in the northern part of the basin. The most significant applied conditioning factors were well density, land subsidence, groundwater drawdown, distance to fault, and geology. The study region increased agricultural and residential areas, and the primary water source is groundwater. For these types of land, groundwater exploitation is exceptionally high, and many unregulated deep wells are mainly used for farming purposes in the Qa’ Jahran Basin. However, the region’s continuous development and urbanization pose significant questions regarding the ability to satisfy future water demand and the resulting dangers of earth fissures. The field is still considered tectonically active, another source and origin of earth fissures. Although this study attempted to incorporate all accessible and relevant data for earth fissure modeling, more factors could be considered, such as sediment thickness and others, which may contribute to better prediction accuracy. On the other hand, the fissure sample was prepared using simply the locations of the fissure events, not the date of the event. However, the timing of the fissuring may represent the effect of the change in some factors, such as land use changes and groundwater fluctuations, on the event of fissuring. This is also an intriguing area for further research. In addition, ML often encounters classification issues. We aim to predict the class label in a classification task by examining the predictor when the target or output variable is categorical. Data imbalance problems may arise in this case and often yield inappropriate results. In the future study, we will discuss the imbalanced dataset, the problem regarding its prediction, and how to deal with such data more efficiently than the conventional ML approaches.

This research provides helpful insights for future studies on detecting earth fissures using ML algorithms. The hazard maps of earth fissures and knowledge of hazardous locations would support decision-makers as a roadmap to make appropriate decisions for handling and tracking the potential losses incurred by the vulnerable environment. The results could also be helpful for water resource managers to enable sound decision-making for groundwater withdrawal regulations. More potential research is required to study the existence of natural hazards.

## Data Availability

The datasets used and/or analyzed during the current study are available from the corresponding author upon reasonable request.

## Code availability

RStudio desktop (version 1.3.9), and R (version 3.6.2) were used for statistical analysis and running the models. All R Studio codes are available upon reasonable request.

## References

[1] Conway BD (2016). Land subsidence and earth fissures in south-central and southern Arizona, USA. Hydrogeol. J..

[2] Wang G (2010). Earth fissures in Jiangsu Province, China and geological investigation of Hetang earth fissure. Environ. Earth Sci..

[3] Xu J (2018). Classification, grading criteria and quantitative expression of earth fissures: a case study in Daming Area, North China Plain. Geomat. Nat. Hazards Risk.

[4] Carpenter MC (1993). Earth-fissure movements associated with fluctuations in ground-water levels near the Picacho Mountains, south-central Arizona, 1980–84.

[5] Holzer TL, Pampeyan EH (1981). Earth fissures and localized differential subsidence. Water Resour. Res..

[6] Holzer, T. L. in *Eighth International Symposium on Land Subsidence.*

[7] Holzer TL, Galloway DL (2005). Impacts of land subsidence caused by withdrawal of underground fluids in the United States. Hum. Geol. Agents.

[8] Holzer TL (1984). Ground failure induced by ground-water withdrawal from unconsolidated sediment. Rev. Eng. Geol..

[9] Leonard R (1929). An earth fissure in southern Arizona. J. Geol..

[10] Lofgren, B. in *Geological Society of America, Abstracts and Programs.*

[11] Pacheco J (2006). Delimitation of ground failure zones due to land subsidence using gravity data and finite element modeling in the Querétaro valley, México. Eng. Geol..

[12] Pacheco-Martínez J (2013). Land subsidence and ground failure associated to groundwater exploitation in the Aguascalientes Valley, México. Eng. Geol..

[13] Li Y, Yang J, Hu X (2000). Origin of ground fissures in the Shanxi Graben system, Northern China. Eng. Geol..

[14] Wang G (2009). Earth fissures triggered by groundwater withdrawal and coupled by geological structures in Jiangsu Province, China. Environ. Geol..

[15] Ye S, Xue Y, Wu J, Yan X, Yu J (2016). Progression and mitigation of land subsidence in China. Hydrogeol. J..

[16] Zhao C (2009). Monitoring of land subsidence and ground fissures in Xian, China 2005–2006: Mapped by SAR interferometry. Environ. Geol..

[17] Gaur V, Kar S, Srivastava M (2015). Development of ground fissures: A case study from southern parts of Uttar Pradesh, India. J. Geol. Soc. India.

[18] Nikbakhti O, Hashemi M, Banikheir M, Basmenj AK (2018). Geoenvironmental assessment of the formation and expansion of earth fissures as geological hazards along the route of the Haram-to-Haram Highway, Iran. Bull. Eng. Geol. Environ..

[19] Ajalloeian R, Ghazifard A, Hashemi M, Kamyab E (2006). Effect of stratigraphy on earth fissuring in the northern Mahyar plain, Iran. Eng. Geol. For Tomorrow’s Cities. Geol. Soc. Lond. Eng. Geol. Spec. Publ..

[20] Youssef AM, Sabtan AA, Maerz NH, Zabramawi YA (2014). Earth fissures in wadi najran, kingdom of saudi arabia. Nat. Hazards.

[21] Khan AS, Khan SD, Kakar DM (2013). Land subsidence and declining water resources in Quetta Valley, Pakistan. Environ. Earth Sci..

[22] Williams F, Williams M, Aumento F (2004). Tensional fissures and crustal extension rates in the northern part of the Main Ethiopian Rift. J. Afr. Earth Sci..

[23] Asfaw LM (1998). Environmental hazard from fissures in the Main Ethiopian Rift. J. Afr. Earth Sci..

[24] Sato C, Haga M, Nishino J (2006). Land subsidence and groundwater management in Tokyo. Intern. Rev. Environ. Strat..

[25] Chiaradonna A, Tropeano G, d’Onofrio A, Silvestri F (2019). Interpreting the deformation phenomena of a levee damaged during the 2012 Emilia earthquake. Soil Dyn. Earthq. Eng..

[26] Ghazifard A, Moslehi A, Safaei H, Roostaei M (2016). Effects of groundwater withdrawal on land subsidence in Kashan Plain, Iran. Bull. Eng. Geol. Environ..

[27] Lee C, Zhang J, Zhang Y (1996). Evolution and origin of the ground fissures in Xian, China. Eng. Geol..

[28] Li X, Wang S, Liu T, Ma F (2004). Engineering geology, ground surface movement and fissures induced by underground mining in the Jinchuan Nickel Mine. Eng. Geol..

[29] Vaz T, Zêzere JL (2016). Landslides and other geomorphologic and hydrologic effects induced by earthquakes in Portugal. Nat. Hazards.

[30] Wan J (2020). Characteristics and main causes of earth fissures in northeastern Beijing Plain, China. Bull. Eng. Geol. Environ..

[31] Elsbury, R. & Van Siclen, D. in *ASCE Convention, Houston, Texas.*

[32] Lee J-Y, Kwon KD, Raza M (2018). Current water uses, related risks, and management options for Seoul megacity, Korea. Environ. Earth Sci..

[33] Ojeda Olivares EA (2019). Climate change, land use/land cover change, and population growth as drivers of groundwater depletion in the Central Valleys, Oaxaca, Mexico. Remote Sens..

[34] Choubin B (2019). Earth fissure hazard prediction using machine learning models. Environ. Res..

[35] Budhu M (2008). Mechanics of earth fissures using the Mohr-Coulomb failure criterion. Environ. Eng. Geosci..

[36] Peng J-B (2013). Physical simulation of ground fissures triggered by underground fault activity. Eng. Geol..

[37] Ye S (2018). A novel approach to model earth fissure caused by extensive aquifer exploitation and its application to the Wuxi case, China. Water Resour. Res..

[38] Zang M, Peng J, Xu N, Jia Z (2021). A probabilistic method for mapping earth fissure hazards. Sci. Rep..

[39] Zhang W (2014). Occurrence assessment of earth fissure based on genetic algorithms and artificial neural networks in Su-Xi-Chang land subsidence area, China. Geosci. J..

[40] Wu Q, Ye S, Wu X, Chen P (2003). A nonlinear modeling and forecasting system of earth fractures based on coupling of artificial neural network and geographical information system—exemplified by earth fractures in Yuci City, Shanxi, China. Environ. Geol..

[41] Jachens RC, Holzer TL (1982). Differential compaction mechanism for earth fissures near Casa Grande, Arizona. Geol. Soc. Am. Bull..

[42] Sheng Z, Helm DC, Li J (2003). Mechanisms of earth fissuring caused by groundwater withdrawal. Environ. Eng. Geosci..

[43] Peng J-B (2017). A proposed solution to the ground fissure encountered in urban metro construction in Xi’an, China. Tunn. Undergr. Space Technol..

[44] Wang Z-F, Shen S-L, Cheng W-C, Xu Y-S (2016). Ground fissures in Xi’an and measures to prevent damage to the Metro tunnel system due to geohazards. Environ. Earth Sci..

[45] Yang C (2018). Deformation at longyao ground fissure and its surroundings, north China plain, revealed by ALOS PALSAR PS-InSAR. Int. J. Appl. Earth Obs. Geoinf..

[46] Howard KW, Zhou W (2019). Overview of ground fissure research in China. Environ. Earth Sci..

[47] Samadianfard S (2019). Support vector regression integrated with fruit fly optimization algorithm for river flow forecasting in Lake Urmia Basin. Water.

[48] Ghamisi P, Plaza J, Chen Y, Li J, Plaza A (2017). Advanced spectral classifiers for hyperspectral images: A review. IEEE Geosci. Remote Sens. Mag..

[49] Chen W (2019). Spatial prediction of landslide susceptibility by combining evidential belief function, logistic regression and logistic model tree. Geocarto Int..

[50] Rahmati O (2019). Land subsidence modelling using tree-based machine learning algorithms. Sci. Total Environ..

[51] Rahmati O (2019). Land subsidence hazard modeling: Machine learning to identify predictors and the role of human activities. J. Environ. Manag..

[52] Oh H-J, Syifa M, Lee C-W, Lee S (2019). Land subsidence susceptibility mapping using bayesian, functional, and meta-ensemble machine learning models. Appl. Sci..

[53] Zhu X, Xu Q, Tang M, Li H, Liu F (2018). A hybrid machine learning and computing model for forecasting displacement of multifactor-induced landslides. Neural Comput. Appl..

[54] Albaroot M, Ahmad A, Al-Areeq N, Sultan M (2016). Tectonostratigraphy of Yemen and geological evolution: A new prospective. Int. J. New Technol. Res. J. Environ. Sci..

[55] Albaroot M, Nabil M, Hamdi S, Mohammed A, Saleh A (2018). Quantification of morphometric analysis using remote sensing and GIS techniques in the Qa’Jahran Basin, Thamar Province, Yemen. Int. J. New Technol. Res..

[56] Bosworth W, Huchon P, McClay K (2005). The red sea and gulf of aden basins. J. Afr. Earth Sci..

[57] Mattash, M. Study of the Cenozoic Volcanics and their associated intrusive rocks in Yemen in relation to rift development. In *Hungarian Acad. Sci* 112 (Eötvös Loránd Univ. Budapest, 1994).

[58] Beydoun Z (1998). International lexicon of stratigraphy. Vol. III Repub. Yemen Second Ed. Int. Union Geol. Sci. Minist. Oil Miner. Resour. Repub. Yemen Publ..

[59] Ullah K, Zhang J (2020). GIS-based flood hazard mapping using relative frequency ratio method: A case study of Panjkora River Basin, eastern Hindu Kush, Pakistan. PLoS ONE.

[60] Mohammady M, Pourghasemi HR, Amiri M (2019). Land subsidence susceptibility assessment using random forest machine learning algorithm. Environ. Earth Sci..

[61] Althuwaynee OF, Pradhan B, Lee S (2016). A novel integrated model for assessing landslide susceptibility mapping using CHAID and AHP pair-wise comparison. Int. J. Remote Sens..

[62] Othman, A. in *Conference of the Arabian Journal of Geosciences.* 287–291 (Springer).

[63] Delgado Blasco JM, Foumelis M, Stewart C, Hooper A (2019). Measuring urban subsidence in the Rome metropolitan area (Italy) with Sentinel-1 SNAP-StaMPS persistent scatterer interferometry. Remote Sens..

[64] Pradhan, B., Seeni, M. I. & Nampak, H. in *Laser Scanning Applications in Landslide Assessment* 69–81 (Springer, 2017).

[65] Bui DT (2016). Hybrid artificial intelligence approach based on neural fuzzy inference model and metaheuristic optimization for flood susceptibilitgy modeling in a high-frequency tropical cyclone area using GIS. J. Hydrol..

[66] Amiri M, Pourghasemi HR, Ghanbarian GA, Afzali SF (2019). Assessment of the importance of gully erosion effective factors using Boruta algorithm and its spatial modeling and mapping using three machine learning algorithms. Geoderma.

[67] Du G-L, Zhang Y-S, Iqbal J, Yang Z-H, Yao X (2017). Landslide susceptibility mapping using an integrated model of information value method and logistic regression in the Bailongjiang watershed, Gansu Province, China. J. Mt. Sci..

[68] Breiman L (2001). Random forests. Mach. Learn..

[69] Lawrence RL, Wood SD, Sheley RL (2006). Mapping invasive plants using hyperspectral imagery and Breiman Cutler classifications (RandomForest). Remote Sens. Environ..

[70] Li X, Cheng X, Chen W, Chen G, Liu S (2015). Identification of forested landslides using LiDar data, object-based image analysis, and machine learning algorithms. Remote Sens..

[71] Karakas G, Can R, Kocaman S, Nefeslioglu H, Gokceoglu C (2020). Landslide susceptibility mapping with random forest model for Ordu, Turkey. Int. Arch. Photogramm. Remote Sens. Spatial Inf. Sci..

[72] Njage PMK, Leekitcharoenphon P, Hald T (2019). Improving hazard characterization in microbial risk assessment using next generation sequencing data and machine learning: predicting clinical outcomes in shigatoxigenic *Escherichia coli*. Int. J. Food Microbiol..

[73] Rahmati O, Pourghasemi HR (2017). Identification of critical flood prone areas in data-scarce and ungauged regions: A comparison of three data mining models. Water Resour. Manag..

[74] Youssef AM, Pourghasemi HR, Pourtaghi ZS, Al-Katheeri MM (2016). Landslide susceptibility mapping using random forest, boosted regression tree, classification and regression tree, and general linear models and comparison of their performance at Wadi Tayyah Basin, Asir Region, Saudi Arabia. Landslides.

[75] Lin X (2010). A random forest of combined features in the classification of cut tobacco based on gas chromatography fingerprinting. Talanta.

[76] Probst P, Wright MN, Boulesteix AL (2019). Hyperparameters and tuning strategies for random forest. Wiley Interdiscip. Rev. Data Min. Knowl. Discov..

[77] Bandara, A. *et al.* A generalized ensemble machine learning approach for landslide susceptibility modeling. In *Data Management, Analytics and Innovation* 71–93 (Springer, 2020).

[78] Wang L, Wang X, Chen A, Jin X, Che H (2020). Prediction of type 2 diabetes risk and its effect evaluation based on the XGBoost model. Healthcare..

[79] Sahin EK (2020). Comparative analysis of gradient boosting algorithms for landslide susceptibility mapping. Geocarto Int..

[80] Sahin EK (2020). Assessing the predictive capability of ensemble tree methods for landslide susceptibility mapping using XGBoost, gradient boosting machine, and random forest. SN Appl. Sci..

[81] Mabdeh, A. N., Al-Fugara, A., Ahmadlou, M. & Pradhan, B. Novel ensemble-based machine learning models based on the bagging, boosting and random subspace methods for landslide susceptibility mapping. Preprint. (2021).

[82] Leung KM (2007). Naive Bayesian classifier. Polytech. Univ. Depart. Comput. Sci./Financ. Risk Eng..

[83] Kelly DL, Kolstad CD (1999). Control Bayesian learning, growth, and pollution. J. Econ. Dyn..

[84] Merghadi A (2020). Machine learning methods for landslide susceptibility studies: A comparative overview of algorithm performance. Earth Sci. Rev..

[85] Zhu R, Hu X, Hou J, Li X, Protection E (2021). Application of machine learning techniques for predicting the consequences of construction accidents in China. Process. Saf..

[86] Abu El-Magd SA, Ali SA, Pham QB (2021). Spatial modeling and susceptibility zonation of landslides using random forest, Naïve Bayes and K-nearest neighbor in a complicated terrain. Earth Sci. Inf..

[87] Kramer O (2013). Dimensionality Reduction with Unsupervised Nearest Neighbors.

[88] Robinson S (2014). Simulation: The Practice of Model Development and Use.

[89] Bui DT, Tuan TA, Klempe H, Pradhan B, Revhaug I (2016). Spatial prediction models for shallow landslide hazards: A comparative assessment of the efficacy of support vector machines, artificial neural networks, kernel logistic regression, and logistic model tree. Landslides.

[90] Walter S (2002). Properties of the summary receiver operating characteristic (SROC) curve for diagnostic test data. Stat. Med..

[91] Bradley AP (1997). The use of the area under the ROC curve in the evaluation of machine learning algorithms. Pattern Recognit..

[92] Saito H, Nakayama D, Matsuyama H (2009). Comparison of landslide susceptibility based on a decision-tree model and actual landslide occurrence: The Akaishi Mountains, Japan. Geomorphology.

[93] Tien Bui D, Pradhan B, Lofman O, Revhaug I (2012). Landslide susceptibility assessment in vietnam using support vector machines, decision tree, and Naive Bayes Models. Math. Probl. Eng..

[94] Guzzetti F, Galli M, Reichenbach P, Ardizzone F, Cardinali M (2006). Landslide hazard assessment in the Collazzone area, Umbria, Central Italy. Nat. Hazards Earth Syst. Sci..

[95] Landis JR, Koch GG (1977). The measurement of observer agreement for categorical data. Biometrics.

[96] Ayalew L, Yamagishi H, Reik G (2004). Ground cracks in Ethiopian Rift Valley: Facts and uncertainties. Eng. Geol..

[97] Murray K, Conner MM (2009). Methods to quantify variable importance: Implications for the analysis of noisy ecological data. Ecology.

[98] Brown S, Nicholls R (2015). Subsidence and human influences in mega deltas: The case of the Ganges–Brahmaputra–Meghna. Sci. Total Environ..

[99] Xu Y-S, Shen S-L, Ren D-J, Wu H-N (2016). Analysis of factors in land subsidence in Shanghai: A view based on a strategic environmental assessment. Sustainability.

[100] Deo RC, Şahin M (2016). An extreme learning machine model for the simulation of monthly mean streamflow water level in eastern Queensland. Environ. Monitor. Assess..

[101] Stamatopoulos C, Petridis P, Parcharidis I, Foumelis M (2018). A method predicting pumping-induced ground settlement using back-analysis and its application in the Karla region of Greece. Nat. Hazards.

[102] Zhang Y, Yu J, Gong X, Wu J, Wang Z (2018). Pumping-induced stress and strain in aquifer systems in Wuxi, China. Hydrogeol. J..

[103] Burbey TJ (2002). The influence of faults in basin-fill deposits on land subsidence, Las Vegas Valley, Nevada, USA. Hydrogeol. J..

[104] Hoque Z (2004). A contingency model of the association between strategy, environmental uncertainty and performance measurement: impact on organizational performance. Int. Bus. Rev..

[105] França S, Cabral HN, Software (2015). Predicting fish species richness in estuaries: Which modelling technique to use?. Environ. Modell..

[106] Araujo MB, Guisan A (2006). Five (or so) challenges for species distribution modelling. J. Biogeogr..

[107] Goetz J, Brenning A, Petschko H, Leopold P (2015). Evaluating machine learning and statistical prediction techniques for landslide susceptibility modeling. Comput. Geosci..

[108] Tella A, Balogun A-L (2021). GIS-based air quality modelling: Spatial prediction of PM10 for Selangor State, Malaysia using machine learning algorithms. Environ. Sci. Pollut. Res.arch.

[109] Costache R (2021). Flash-flood potential index estimation using fuzzy logic combined with deep learning neural network, naïve Bayes, XGBoost and classification and regression tree. Geocarto Int..

[110] Mirzaei S, Vafakhah M, Pradhan B, Alavi SJ (2021). Flood susceptibility assessment using extreme gradient boosting (EGB), Iran. Earth Sci. Inform..

[111] Naghibi SA, Vafakhah M, Hashemi H, Pradhan B, Alavi SJ (2020). Water resources management through flood spreading project suitability mapping using frequency ratio, k-nearest neighbours, and random forest algorithms. Nat. Resour. Res..

[112] Meliho M, Khattabi A, Asinyo J (2021). Spatial modeling of flood susceptibility using machine learning algorithms. Arab. J. Geosci..

[113] Madhuri R, Sistla S, Srinivasa Raju K, Change C (2021). Application of machine learning algorithms for flood susceptibility assessment and risk management. J. Water.

[114] Elith J, Leathwick JR, Hastie T (2008). A working guide to boosted regression trees. J. Anim. Ecol..

[115] Mellor A, Boukir S, Haywood A, Jones S (2015). Exploring issues of training data imbalance and mislabelling on random forest performance for large area land cover classification using the ensemble margin. ISPRS J. Photogramm. Remote Sens..

